# Root cause analysis of safety incidents in antineoplastic use in children

**DOI:** 10.1590/0034-7167-2021-0139

**Published:** 2024-07-15

**Authors:** Elizangela Domiciano Garcia Barreto, Valcieny Souza Sandes, Gustavo Cattelan Nobre, Monica Martins, Sima Esther Ferman, Elisangela Costa Lima

**Affiliations:** I Universidade Federal do Rio de Janeiro. Rio de Janeiro, Rio de Janeiro, Brazil; II Instituto Nacional de Câncer. Rio de Janeiro, Rio de Janeiro, Brazil; III Escola Nacional de Saúde Pública Sergio Arouca- Fiocruz. Rio de Janeiro, Rio de Janeiro, Brazil

**Keywords:** Root Cause Analysis, Antineoplastic Agents, Patient Safety, Medication Errors, Child, Análisis de Causa Raíz, Antineoplásicos, Seguridad del Paciente, Errores de Medicación, Niño

## Abstract

**Objectives:**

to identify and analyze the factors that contribute to safety incident occurrence in the processes of prescribing, preparing and dispensing antineoplastic medications in pediatric oncology patients.

**Methods:**

a quality improvement study focused on oncopediatric pharmaceutical care processes that identified and analyzed incidents between 2019-2020. A multidisciplinary group performed root cause analysis (RCA), identifying main contributing factors.

**Results:**

in 2019, seven incidents were recorded, 57% of which were prescription-related. In 2020, through active search, 34 incidents were identified, 65% relating to prescription, 29% to preparation and 6% to dispensing. The main contributing factors were interruptions, lack of electronic alert, work overload, training and staff shortages.

**Conclusions:**

the results showed that adequate recording and application of RCA to identified incidents can provide improvements in the quality of pediatric oncology care, mapping contributing factors and enabling managers to develop an effective action plan to mitigate risks associated with the process.

## RESUMEN

**Objetivos:**

identificar y analizar los factores que contribuyen a la ocurrencia de incidentes de seguridad en los procesos de prescripción, preparación y dispensación de medicamentos antineoplásicos en pacientes pediátricos con cáncer.

**Métodos:**

estudio de mejora de la calidad centrado en los procesos de atención farmacéutica oncopediátrica que identificó y analizó incidencias entre 2019-2020. Un grupo multidisciplinario realizó un análisis de causa raíz (ACR), identificando los factores contribuyentes clave.

**Resultados:**

en 2019 se registraron siete incidentes, el 57% relacionados con la prescripción. En 2020, mediante búsqueda activa se identificaron 34 incidencias, el 65% relacionadas con la prescripción, el 29% con la preparación y el 6% con la dispensación. Los principales factores contribuyentes fueron las interrupciones, la falta de alerta electrónica, la sobrecarga de trabajo, la capacitación y la escasez de personal.

**Conclusiones:**

los resultados mostraron que el registro adecuado y la aplicación del ACR a los incidentes identificados pueden proporcionar mejora de la calidad de la atención del cáncer pediátrico mediante el mapeo de los factores contribuyentes y permitiendo a los administradores desarrollar un plan de acción eficaz para mitigar los riesgos asociados con el proceso.

## INTRODUCTION

Cancer is the leading cause of death from disease in children and adolescents in developed countries. In Brazil, an estimated 7,930 new cases per year^(1)^. Despite technological and scientific advances, safe, effective and individualized care is still a challenge for continuous quality improvement in pediatric oncology^(2-3)^. The estimated risk of significant harm to patients due to failures in the processes involving treatment varies from one to four for every 1,000 medications prescribed^(4-5)^. With multifaceted dosage schemes, chemotherapy requires antineoplastic drugs classified as potentially dangerous, which require high vigilance, given their toxicity and narrow therapeutic indices, even within recommended doses^(6)^.

Evidence has shown that 10% of children with leukemia or lymphoma experience adverse drug events due to outpatient medication errors^(7)^. The rate of medication errors resulting in harm or death in children (31%) is almost three times that of adults (13%)^(8)^.

A study carried out in a chemotherapy unit for pediatric patients in Mexico identified that, of all medication errors, 37.2% had the potential to cause harm to children^(9)^. Factors were associated with (i) the calculation of individualized doses according to age, weight (mg/kg) and body surface area (mg/m^2^), which involve multiple mathematical operations at various steps of the medication process; (ii) the need to dilute the medication to administer small doses; (iii) the immature liver and kidney systems involved in drug metabolism; and (iv) children’s inability to report side effects increases the vulnerability of these patients to adverse events^(8,10-11)^. Brazilian studies on medication errors in pediatric oncology are scarce^(12)^. A study in a Brazilian pediatric ward identified problems in 5.3% of prescriptions analyzed over two years^(11)^.

Patient safety, in addition to seeking to reduce adverse event occurrence with harm, is also concerned with other incidents that have the potential to cause harm, but that did not affect patients^(13)^. The success of pediatric oncology care depends on teamwork, carried out through continuous communication, since this care process is complex and measuring the quality of care refers to different dimensions^(2-3,14)^. Using appropriate tools is essential to identify, analyze contributing factors and propose solutions to observed problems and incidents^(15)^. Among these, root cause analysis (RCA) is a systematic, relatively simple investigation strategy that seeks to understand the causes of the incident^(16-17)^.

RCA is a method that leads to a greater understanding of the cause of the accident, with less focus on the individual who committed the error and more information on organizational factors. It helps to identify the reason for the occurrence, and not just describe the event^(17-18)^. It is a low-cost strategy that helps mitigate medication errors. Knowing its application can be useful to increase the safety of the treatment of pediatric cancer patients, as it allows mapping the types of incidents related to the processes of prescription, preparation and dispensing of antineoplastic drugs in pediatrics and analyzing the contributing factors, identifying weaknesses in the process and to outline effective action plans to improve the quality of care for these patients.

## OBJECTIVES

To identify and analyze the factors that contributed to safety incident occurrence in the steps of prescribing, preparing and dispensing antineoplastic medications for pediatric patients in a national reference hospital for oncology treatment.

## METHODS

### Ethical aspects

The study was approved by the Research Ethics Committee of the study institution. The professionals invited and who agreed to participate in the study were previously informed about the objectives and methodological procedures. Participation was confirmed by signing the Informed Consent Form.

### Study design, period and location

Single-case quality improvement study that focused on pharmaceutical care processes in the pediatric oncology and pediatric hematology sections of a federal hospital of national reference for oncology treatment, located in the city of Rio de Janeiro, in January 2019 to December 2020. Djulbegovic^(19)^ conceptualizes Health Care Improvement Science as “a framework for research focused on improving healthcare”. This design can use different approaches with the aim of providing an improvement to a problem defined in a given location^(20)^.

The Standards for Quality Improvement Reporting Excellence (SQUIRE 2.0) was used as a reference for study description. This instrument adapts to many types of projects, including those that aim to understand how a local process works, seeking to improve health care quality and safety. Each SQUIRE 2.0 item was assessed for relevance to the present study and applied whenever possible, taking into account that some items do not apply to the present study^(21)^.

### Population; inclusion and exclusion criteria

The study population was made up of patients aged zero to 19 years undergoing treatment with intravenous antineoplastic therapy and who recorded incident occurrence as described in the study protocol during the studied period. Patients exclusively using antineoplastics administered by other routes were excluded from analysis.

### Study protocol

**Figure 1:**
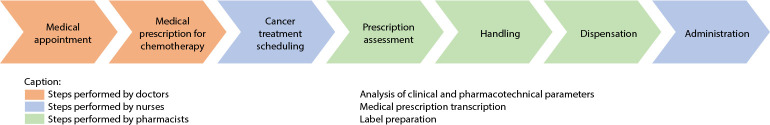
Flowchart of the steps of care in the reference center studied, Rio de Janeiro, Rio de Janeiro, Brazil, 2022

In the pharmaceutical assistance process, three steps were selected for this study, such as antineoplastic medication prescription, preparation and dispensing (Figure 1). After medical consultation, treatment prescription was attached to the medical record and forwarded to nursing who, after preliminary checking of prescription and treatment, scheduled the date for medication administration.

After this step, the medical record was sent to pharmacy department for validity by a pharmacist. This professional assessed name, registration number, weight, height, body surface, age, sex, physician’s name, signature and stamp with registration number with the class council. Then, clinical aspects such as results of laboratory assessments, possible toxicities, disease staging, dosage to be administered per time interval, routes of administration and therapeutic plan were also considered. Finally, pharmacotechnical issues (compatibility, diluent, concentration) were analyzed. Then, medical prescription was transcribed into a Microsoft Excel^®^ spreadsheet that guided antineoplastic drug preparation and dispensing to enable the return of the medical record. Any incidents involving this process were also recorded on the same spreadsheet. Incident, as defined in Ministerial Ordinance 529 of 2013, it is “an event or circumstance that could have resulted, or resulted, in unnecessary harm to patients”^(22)^.

After preparation, medication was labeled, with the label and product data checked with the prescription transcribed in the day’s diary for later forwarding to the nursing team. Nursing is responsible for the last phase of this process, checking the label with medication prescription by two nurses and identifying patients before administration.

Data collection was carried out in two phases. The first phase consisted of retrospectively collecting data on incidents reported to the Patient Safety Center between January and December 2019.

In the second phase (January to December 2020), there was an active search for incidents and an incentive for professionals involved in the assistance process to adhere to report. The instrument used to collect information was a spreadsheet that was already usually adopted to record scheduled patients. A column called “Intervention” was created, where the pharmacist freely described the incident and the outcome. At this stage, prospective data was collected on incidents that occurred between January and December 2020, and the analyzed processes were maintained in accordance with institutional routine. As this was a prospective phase, the problems and incidents identified were immediately corrected by the team. Additional information for a complete description of the recorded incident (team involved, stage of the process performed and any people affected) was collected from patients’ medical record.

### Organization of information and root cause analysis

Each incident was classified according to the step of the process: prescription, preparation and dispensing. It was observed that the description of some incidents was similar, which led to them being grouped together. All collected data was organized in a Microsoft Excel^®^ spreadsheet.

For RCA, three meetings were held with professionals with experience in pediatric oncology who participated in the analysis group. The first meeting, a virtual meeting, using Google Meet^®^, lasting 120 minutes, was attended by professionals with experience in oncology (nurses, physicians, pharmacists and an IT technician responsible for developing the management system integrated system used in the institution). There was a discussion, in general terms, of identified incidents and the associated care processes. The focus of the meeting was incident description and understanding, but possible improvement actions were also highlighted and recorded.

Subsequently, two face-to-face meetings, lasting 90 minutes, were organized to discuss incidents related to the preparation and dispensing processes, with the presence of five pharmacists and three pharmacy technicians and related to medical prescription, with two physicians and two pharmacists.

At each meeting, participants used the brainstorming technique, which is a round of ideas in a short period, aimed at searching for suggestions through group work. The use of this method is based on the assumption that a group is capable of having more ideas than isolated individuals. Its purpose is to provide maximum information through the knowledge of members involved in the subject, seeking to find the possible causes of a given problem^(23)^. Ideas were systematized using the five whys method, guided by the question “Why did this incident occur?”. With each answer described, the question was asked why the previous statement was true, until the answers stopped, suggesting the identification of the root cause. Based on the responses reported by participants, the cause and effect diagram (Ishikawa or fishbone diagram) was used to explore and indicate the possible causes of the incidents analyzed^(16-17,24)^. In these two meetings, the application of RCA made it possible to organize, classify, document and graphically display the causes of incidents, grouped by categories for discussion and analysis by participants^(17)^.

## RESULTS

During the 24-month study period, 41 incidents were recorded by pharmacists and nurses. In 2019, 281 patients were treated, generating 5127 consultations, and seven incidents were reported for six patients. One incident was related to the dispensing process (14%), two to the preparation process (29%) and four to the prescription process (57%), one of which hit the patient but did not cause harm, resulting in the administration of an overdose of chemotherapy.

In 2020, 280 patients were treated, with 4,897 consultations carried out, and 34 incidents were reported for 30 patients through active search, after monitoring spreadsheet implementation. No incidents affected the patient, demonstrating the effectiveness of the barriers present. Of the 34 reports, two incidents were dispensing (6%), 10 were preparation (29%), and 22 were prescription (65%).

Incidents involving the medical prescription of chemotherapy were the most prevalent. Of the 26 reports during the study period, 50% were manual prescriptions, 26.9% electronic prescriptions, and 23.1% electronic prescriptions with manual change. The 22 records from 2020 were equivalent to 1.4% of prescriptions analyzed.

Chart 1 summarizes the main information on the incidents found and contributing factors identified by the multidisciplinary group that analyzed the incidents; in this case, the most common medication prescribed on a different day or dose than the therapeutic protocol stands out, involving four medications.

**Chart 1:** Incidents involving injectable antineoplastic medication and contributing factors in the prescription, preparation and dispensing processes in the reference center studied (n=41), Rio de Janeiro, Rio de Janeiro, Brazil, 2019-2020

Type	Description	N	Contributing Factors	Medications Involved
Prescription	Medication prescribed on a different day or dose than the therapeutic protocol	7	Manual prescription Interruptions Lack of electronic prescription for some protocols	Asparaginase, carboplatin Cyclophosphamide, vincristine
	Adjusting the formula by weight for patients weighing less than 10 or 12 kg	5	Absence of electronic alert (barrier)	Actinomycin#, carboplatin, etoposide, ifosfamide
	Supportive medication omission	5	Lack of policy for including supportive medications Lack of permanent education for professionals	Diphenhydramine, electrolytes, hydrocortisone, ondansetron
	Mistake in dose calculation	4	Absence of electronic prescription Lack of attention	Cyclophosphamide, electrolytes
	Need for dose reduction	3	Absence of electronic alert (barrier) Team overload	Carboplatin, irinotecan, mesna
	Dose omission	1	Absence of electronic prescription Lack of attention	Cytarabine
	Presence of doubtful information	1	Lack of knowledge of registered protocols	Cyclophosphamide
Preparation	Request to prepare the medication with a different dose	5	Mistake in transcription Lots of interruptions Failure to check the compounded medication with the prescription	Carboplatin, etoposide, ifosfamide, mesna, vincristine
	Wrong request to prepare unprescribed medication	4	Error in transcription High number of prescriptions to analyze Many interruptions Failure to check the compounded medication with the prescription Multiple tasks Delay in moving medical records	Carboplatin, Doxorubicin, vincristine
	Wrong identification on the vial of products handled in the biological safety cabinet	2	Failure to check the label with the handled bag Prescription delivery times at the pharmacy Number of patients Limited physical space Employee deficit Work overload Tiredness of employees who spend a long time in the handling area	Cyclophosphamide/ifosfamide Cyclophosphamide/cytarabine
	Wrong labeling by the technician	1	Failure to check the label with the handled bag Employee deficit Work overload Tiredness of employees who spend a long time in the handling area	Doxorubicin
Dispensation	Omission of prescribed medication	2	Error in transcription Failure to check compounded medication with prescription Multiple tasks	Vincristine, mesna
	Medication dispensed to the wrong patient	1	Limited physical space Lack of adequate packaging material Hand label with illegible handwriting	Ondansetron

# *- incident that affected the patient; kg - kilograms.*

**Figure 2:**
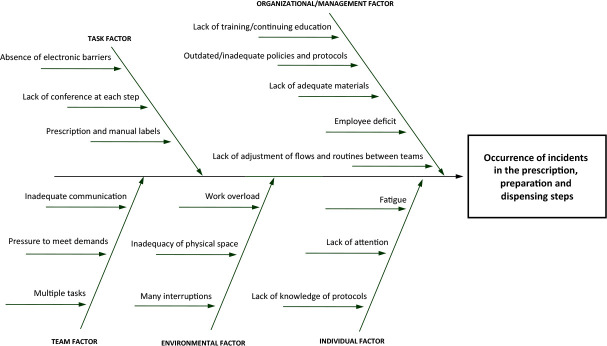
Cause and effect diagram of incidents involving injectable antineoplastic medication in the reference center studied, Rio de Janeiro, Rio de Janeiro, Brazil, 2019-2020

The main contributing factors mentioned by participants, for the three steps assessed, were grouped in Figure 2. Thus, they were related to organizational and individual aspects, in addition to the task, the environment and the team.

## DISCUSSION

This study allowed the team responsible for pharmaceutical assistance in pediatric oncology and hematology sections to identify and analyze incidents related to using antineoplastic drugs, improving quality of care in the studied hospital. Furthermore, it allowed understanding the complexity and scope of medication errors by identifying microprocesses and interconnected and interrelated activities involving the entire multidisciplinary team, carried out by different professionals. Furthermore, the analytical tools used can potentially be applied to other problems in quality of care, patient safety and other issues in the organization’s management. It is worth highlighting the merit of collectively involving the team of professionals and thus improving communication and strengthening the organizational culture and in particular safety culture^(25)^.

One of the main pillars for an effective program in this area are voluntary report systems, as they can produce useful and strategic information to guarantee quality and foster a safety culture. For this to occur, such systems need to be widely used among professionals. In this regard, it is understood that underreporting of incidents compromises patient safety and is a major challenge for institutions^(26-27)^. The present study was able to assess the extent to which incidents can be underreported. A significant difference was observed between the data collection phases. The difference in reports was almost five times greater in the second phase (active search), where there was an incentive and stimulus for reports.

The low rate of reports in the period of 2019 corroborates what is described in the literature, which points out that a high percentage of professionals do not report events or incidents related to patient safety^(28)^. The lack of report, even when the incident does not affect patients, deprives the health service of carrying out an adequate analysis of contributing factors, promoting assertive interventions and improving patient care quality. Bião and Silva^(28)^ looked at the patient safety culture in a large Brazilian hospital organization and, using interviews with health professionals, found that 87.8% of professionals did not report any event in 12 months. These findings suggest that professionals do not report incidents for fear of reprimand, punishment and humiliation, or because of disbelief that reporting will not lead to any change; thus, they reinforce that a fair safety culture is not completely embedded in healthcare organizations^(29-30)^.

Prescribing is a critical step in the medication process, and a dose error at this step carries a high risk of causing harm to patients. Prescribing errors can be avoided prior to administration if appropriate safety structures are in place. Standardization of processes, improvement and emphasis on the education of prescribing professionals and expansion of clinical pharmacists’ role for better health team integration are improvement measures identified as factors that minimize errors in prescriptions^(31-33)^. The frequency of prescription errors in 2020 was 1.4%, and this falls within the range reported in international literature, which varies between 0.1% and 24.6%^(34)^. Another study found that the most frequently encountered medication error was prescription error (42.31%), followed by administration error (37.18%)^(35)^. The main drugs involved were cyclophosphamide, cytarabine, doxorubicin, etoposide, ifosfamide, irinotecan and vincristine. In the literature, the most commonly reported medications were methotrexate (15%), cytarabine (12%), and etoposide (8%)^(32)^.

The hospital’s prescription management computer program, developed in-house, has the capacity to delete handwritten prescriptions. The implementation of electronic prescription systems represents a major advance in the strategies used to minimize errors arising from prescriptions, but for this to occur, this measure must be adopted by the entire medical team. The way prescriptions were written (handwritten or electronic) was related to the incidents observed, since among all recorded prescription incidents, and 73% involved manual or electronic prescriptions with some manual change. Albarrak *et al*.^(36)^ observed that the distribution of errors between handwritten and electronic prescriptions was 35.7% and 2.5%, respectively. Studies show that electronic prescription can reduce medication error occurrence, improving prescription quality and patient safety^(37)^.

Some prescription incidents could be avoided by barriers, such as issuing automatic and appropriate alerts during the electronic prescription process, assisting and supporting physicians in clinical decisions. Adhering to computerized tools for prescription is strategic for a safe process, and it is essential to verify the system operational characteristics, as the existence of a program for prescribing medications does not accredit the process as safe^(38-39)^.

Reinhardt *et al*.^(31)^ report that technological improvement favors a potential increase in safety in the use of chemotherapy, but even with modifications to the computer program, around 39% of errors would remain unidentified and uncorrected by the computerized system. Therefore, the professionals involved in the chain continue to be of fundamental importance in intercepting such errors, thus contributing to improving the safety of this high-risk patient population.

Based on the premise that the occurrence of a medication error can occur at any stage of the process, and that it is based on the decisions and actions of different professionals with different backgrounds and behaviors, it is indisputable to raise awareness among professionals who are likely to make errors. In this awareness raising, the need for understanding and assimilation of the negative impacts on the process and risks for patients is emphasized.

An integrative review identified that the main risk factors related to intravenous medication preparation were psychological aspects and work overload, factors related to the work environment and the lack of updating in health education^(40)^. Another cross-sectional descriptive study recruited nurses from three tertiary hospitals in South Korea. Data collected via structured questionnaires showed that greater patient safety competencies were directly associated with professionals who work less than 40 hours per week, while those who work more than 50 hours weekly presented more adverse results, having detrimental effects on patient safety^(41)^.

The various interruptions during the execution of their tasks were also a critical point raised in the present study. These interruptions tend to be frequent and rooted in the culture of health institutions. Most professionals are condescending, as they consider this problem as something inherent to the work^(42)^. This scenario favorable to incident occurrence in care, which impact the accuracy of the activities carried out, was attributed as a contributing factor to incident occurrence. According to the recommendations of the Institute for Safe Practices in Using Medications^(42-43)^, it is essential to plan strategies to avoid interruptions in activities during the medication process. It is worth highlighting that the strategies will be more satisfactory if accompanied by an educational approach that promotes awareness among professionals and changes in culture in the institution. From another perspective, teams with a reduced and inadequate number of professionals, when compared to the number of patients receiving care, are also more likely to be interrupted, as they appear more exhausted, hasty and require multiple tasks^(42)^.

In this context, human resources management plays a fundamental role in health services, balancing the institution’ needs with appropriate use of financial resources and professionals’ and patients’ well-being, directly affecting the quality of services provided and the degree of satisfaction of users^(44)^. Health units must have an adequate number of professionals, providing the development of safe processes without occupational overload, respecting the legally established weekly working hours limit and current legislation^(42,44)^.

It is important to highlight that only one of the recorded incidents reached patients and, despite the potential, did not cause serious harm, as patients were monitored after the event was identified. This reflects the importance of collaborative care carried out by the multidisciplinary team in promoting quality and patient safety. Watts and Parsons^(45)^ showed that a multidisciplinary team working on a prospective pharmaceutical surveillance system for chemotherapy prescription and administration errors managed to reduce by half error occurrence observed in pediatric oncology. Thus, in our understanding, this study reinforces (i) the importance of microprocesses that involve pharmaceutical care and (ii) how a multidisciplinary and thorough analysis can reveal ways to improve quality of care.

### Study limitations

This study has limitations, among which the single case study design stands out, which focused on the experience of a reference hospital in pediatric oncology. The number of meetings held (three) proved to be adequate for the proposal, but it is possible that increasing the number of meetings could have led to greater exploration and exhaustive discussion of incidents found, an aspect not assessed here, as it focused on contributing factors. It is known that RCA is an easy-to-use strategy in hospitals, useful for identifying remote and immediate causes of security incidents; however, the effectiveness in implementing measures to prevent problems involves other elements of the internal and external organizational contexts^(46)^.

### Contributions to nursing, health and public policies

Given the lack of studies on pediatric care, a strong point of this study is the possibility of using the analyzed data to compare results with other institutions and raise awareness among professionals regarding safety culture in using antineoplastic medications, considering that the methodology used could be adapted to support the same analysis in other institutions.

## CONCLUSIONS

The findings highlighted the magnitude of prescription incident occurrence, followed by occurrences in medication preparation. It was identified that the main factors that contributed to the occurrence of safety incidents were the absence of conference and electronic barriers, preparation of manual prescriptions and labels, organizational and environmental issues, communication problems, work overload, fatigue and lack of attention The results presented show that a relatively simple intervention, such as adequate RCA recording and application to identified incidents, can provide an improvement in quality of care in pediatric oncology, mapping contributing factors and enabling managers to develop an effective action plan to mitigate risks associated with the steps analyzed.

It is expected that the experience described in RCA application and the results obtained can serve as subsidies to improve patient safety, encouraging and guiding managers, authorities and health professionals with regard to proposing initiatives and strategies that better respond to institutions’ specific needs. The implementation of systematic safe processes in medication use does not represent a guarantee of safety and quality of health care only for patients, as it also means the protection of the professionals and institutions involved.

## Data Availability

*https://doi.org/10.48331/scielodata.ZOQGJE*
