# Supplementary material for: Root cause analysis of safety incidents in antineoplastic use in children
Source: Rev Bras Enferm. 2024 Jul 15;77(Suppl 3):e20230139. doi: 10.1590/0034-7167-2021-0139 (PMC11253835; doi:10.1590/0034-7167-2021-0139)
Supplement: Translation

# Translation: Análise de causa raiz de incidentes de segurança na utilização de antineoplásicos em crianças

**DOI:** 10.1590/0034-7167-2021-0139pt

**Authors:** Elizangela Domiciano Garcia Barreto, Valcieny Souza Sandes, Gustavo Cattelan Nobre, Monica Martins, Sima Esther Ferman, Elisangela Costa Lima

**Affiliations:** I Universidade Federal do Rio de Janeiro. Rio de Janeiro, Rio de Janeiro, Brasil; II Instituto Nacional de Câncer. Rio de Janeiro, Rio de Janeiro, Brasil; III Escola Nacional de Saúde Pública Sergio Arouca- Fiocruz. Rio de Janeiro, Rio de Janeiro, Brasil

**Keywords:** Análise de Causa Raiz, Antineoplásicos, Segurança do Paciente, Erros de Medicação, Criança

## Abstract

**Objetivos:**

identificar e analisar os fatores contribuintes para ocorrência de incidentes de segurança nos processos de prescrição, preparo e dispensação de medicamentos antineoplásicos em pacientes oncopediátricos.

**Métodos:**

estudo de melhoria da qualidade focado nos processos de assistência farmacêutica oncopediátrica que identificou e analisou incidentes entre 2019-2020. Um grupo multidisciplinar realizou análise de causa raiz (ACR), identificando principais fatores contribuintes.

**Resultados:**

em 2019, registraram-se sete incidentes, sendo 57% relacionados à prescrição. Em 2020, através de busca ativa, identificaram-se 34 incidentes, sendo 65% relativos à prescrição, 29% ao preparo e 6% à dispensação. Os principais fatores contribuintes foram interrupções, ausência de alerta eletrônico, sobrecarga de trabalho, treinamento e déficit de funcionários.

**Conclusões:**

os resultados mostraram que registro adequado e aplicação da ACR aos incidentes identificados podem proporcionar melhoria na qualidade do cuidado oncopediátrico mapeando os fatores contribuintes e possibilitando aos gestores desenvolverem plano de ação efetivo para mitigar riscos associados ao processo.

## INTRODUÇÃO

O câncer é a principal causa de morte por doenças em crianças e adolescentes em países desenvolvidos. No Brasil, estimam-se 7.930 novos casos por ano^(1)^. Apesar dos avanços tecnológicos e científicos, o cuidado seguro, efetivo e individualizado ainda é um desafio para melhoria contínua da qualidade em oncopediatria^(2-3)^. O risco estimado de ocorrência de danos significativos ao paciente em decorrência de falhas nos processos que envolvem o tratamento varia de um a quatro para cada 1.000 medicamentos prescritos^(4-5)^. Com esquemas posológicos multifacetados, a quimioterapia demanda medicamentos antineoplásicos classificados como potencialmente perigosos que pressupõem alta vigilância, visto sua toxidade e índices terapêuticos estreitos, mesmo dentro das doses recomendadas^(6)^.

Evidências demonstraram que 10% das crianças com leucemia ou linfoma sofreram eventos adversos a medicamentos devido a erros de medicação ambulatorial^(7)^. A taxa de erros de medicação que resulta em danos ou morte em crianças (31%) é quase três vezes maior que a dos adultos (13%)^(8)^.

Estudo realizado em uma unidade de quimioterapia para pacientes pediátricos no México identificou que, de todos os erros de medicação, 37,2% possuíam um potencial de causar lesão em crianças^(9)^. Fatores foram associados (i) ao cálculo de doses individualizadas conforme idade, peso (mg/kg) e área de superfície corporal (mg/m^2^), que envolvem múltiplas operações matemáticas em várias fases do processo de medicação; (ii) à necessidade de diluição do medicamento para administrar pequenas doses; (iii) aos sistemas hepáticos e renais imaturos envolvidos no metabolismo do medicamento; e (iv) à incapacidade da criança comunicar efeitos colaterais, aumentando a vulnerabilidade desses pacientes aos eventos adversos^(8,10-11)^. Estudos brasileiros acerca dos erros de medicação em oncopediatria são escassos^(12)^. Estudo em uma enfermaria pediátrica brasileira identificou problemas em 5,3% das prescrições analisadas ao longo de dois anos^(11)^.

A segurança do paciente, além de buscar reduzir a ocorrência de eventos adversos com dano, preocupa-se também com outros incidentes com potencial de causar dano, mas que não atingiram o paciente^(13)^. O sucesso do cuidado oncológico pediátrico depende do trabalho em equipe, realizado por meio de comunicação contínua, uma vez que esse processo de cuidado é complexo e que a mensuração da qualidade do cuidado remete-se a distintas dimensões^(2-3,14)^. O uso de ferramentas adequadas é imprescindível para identificar, analisar os fatores contribuintes e propor soluções para os problemas e incidentes observados^(15)^. Entre essas, a análise de causa raiz (ACR) é uma estratégia de investigação sistemática relativamente simples que busca o entendimento das causas do incidente^(16-17)^.

A ACR é um método que leva a um maior entendimento da causa do acidente, com menor foco no indivíduo que cometeu o erro e mais informações sobre fatores organizacionais. Ela auxilia na identificação do motivo de ocorrência, e não apenas na descrição do evento^(17-18)^. É uma estratégia de baixo custo que auxilia na mitigação de erros de medicação. Conhecer sua aplicação pode ser útil para aumentar a segurança do tratamento de pacientes oncopediátricos, pois permite mapear os tipos de incidentes relacionados aos processos de prescrição, preparo e dispensação de medicamentos antineoplásicos em pediatria e analisar os fatores contribuintes, identificando as fragilidades do processo e para delinear planos de ação efetivos para melhoria da qualidade do cuidado desses pacientes.

## OBJETIVOS

Identificar e analisar os fatores que contribuíram para a ocorrência de incidentes de segurança nas etapas de prescrição, preparo e dispensação de medicamentos antineoplásicos para pacientes pediátricos em hospital de referência nacional para tratamento oncológico.

## MÉTODOS

### Aspectos éticos

O estudo foi aprovado pelo Comitê de Ética em Pesquisa da instituição do estudo. Os profissionais convidados e que aceitaram participar do estudo foram previamente esclarecidos sobre os objetivos e procedimentos metodológicos. A participação foi firmada através da assinatura do Termo de Consentimento Livre e Esclarecido.

### Desenho, período e local do estudo

Estudo de melhoria da qualidade de caso único, que focou nos processos de assistência farmacêutica nas seções de oncologia pediátrica e de hematologia pediátrica de um hospital federal de referência nacional para o tratamento oncológico, localizado no município do Rio de Janeiro, no período de janeiro de 2019 a dezembro de 2020. Djulbegovic^(19)^ conceitua a Ciência da Melhoria do Cuidado de Saúde como “uma estrutura para pesquisa focada na melhoria dos cuidados de saúde”. Esse tipo de estudo pode utilizar diferentes abordagens com o objetivo de proporcionar uma melhoria para um problema definido em determinado local^(20)^.

O instrumento *Standards for Quality Improvement Reporting Excellence* (SQUIRE 2.0) foi utilizado como referencial para a descrição do estudo. Esse instrumento se adapta a muitos tipos de projetos, inclusive os que visam conhecer como funciona um processo local, buscando sua melhoria, qualidade e segurança dos cuidados de saúde. Cada item do SQUIRE 2.0 foi avaliado quanto à relevância para o presente estudo, e aplicado sempre que possível, levando em consideração que alguns itens não se aplicam ao presente estudo^(21)^.

### População; critérios de inclusão e exclusão

A população de estudo foi composta por pacientes de zero a 19 anos de idade em tratamento com terapia antineoplásica intravenosa e que registraram a ocorrência de incidentes segundo descrito no protocolo de estudo no período estudado. Foram excluídos da análise os pacientes em uso exclusivo de antineoplásicos administrados por outras vias.

### Protocolo de estudo

No processo de assistência farmacêutica, três etapas foram selecionadas para o presente estudo: prescrição, preparo e dispensação do medicamento antineoplásico (Figura 1). Após consulta médica, a prescrição do tratamento era anexada ao prontuário e encaminhada para a enfermagem que, após conferência preliminar da prescrição e tratamento, agendava a data a ser realizada a administração dos medicamentos.

**Figura 1:** Fluxograma das etapas do cuidado no centro de referência estudado, Rio de Janeiro, Rio de Janeiro, Brasil, 2022

Após essa etapa, o prontuário era encaminhado ao setor de farmácia para a validação pelo farmacêutico. Esse profissional avaliava nome, matrícula, peso, altura, superfície corporal, idade, sexo, nome do médico, assinatura e carimbo com número de registro no conselho de classe. Em seguida, aspectos clínicos, como resultados de avaliações laboratoriais, possíveis toxicidades, estadiamento da doença, dosagem a ser administrada por intervalo de tempo, vias de administração e plano terapêutico também, eram considerados. Por fim, questões farmacotécnicas (compatibilidade, diluente, concentração) eram analisadas. Em seguida, a prescrição médica era transcrita para uma planilha do *Microsoft Excel* ^®^, que orientava o preparo e a dispensação de antineoplásicos para possibilitar a devolução do prontuário. Quaisquer incidentes envolvendo esse processo também eram registrados na mesma planilha. Incidente, como definido na Portaria Ministerial nº 529 de 2013, é *“*evento ou circunstância que poderia ter resultado, ou resultou, em dano desnecessário ao paciente”^(22)^.

Após o preparo, o medicamento era rotulado, com a conferência dos dados do rótulo e do produto com a prescrição transcrita na agenda do dia para posterior encaminhamento à equipe de enfermagem. A enfermagem é responsável pela última fase desse processo, ocorrendo a conferência do rótulo com prescrição da medicação por dois enfermeiros e identificação do paciente antes da administração.

A coleta de dados foi realizada em duas fases. A primeira fase consistiu na coleta retrospectiva de dados sobre os incidentes notificados ao Núcleo de Segurança do Paciente no período entre janeiro e dezembro de 2019.

Na segunda fase (janeiro a dezembro de 2020), houve uma busca ativa dos incidentes e um incentivo à adesão de notificação pelos profissionais envolvidos no processo de assistência. O instrumento utilizado para a coleta de informações foi uma planilha que já era usualmente adotada para registro dos pacientes agendados. Houve a criação de uma coluna denominada “Intervenção”, onde o farmacêutico livremente descreveu o incidente e o desfecho. Nessa etapa, foram coletados dados prospectivos sobre incidentes ocorridos entre janeiro e dezembro de 2020, e os processos analisados foram mantidos conforme a rotina institucional. Por se tratar de uma fase prospectiva, os problemas e incidentes identificados foram imediatamente corrigidos pela equipe. Informações adicionais para completa descrição de incidente registrado (equipe envolvida, etapa do processo executado e eventuais pessoas afetadas) foram coletadas no prontuário do paciente.

### Organização das informações e análise de causa raiz

Cada incidente foi classificado quanto à etapa do processo: prescrição, preparo e dispensação. Observou-se que a descrição de alguns incidentes era similar, o que levou a um agrupamento deles. Todos os dados coletados foram organizados em uma planilha do programa *Microsoft Excel* ^®^.

Para a ACR, foram realizadas três reuniões com profissionais com experiência em oncopediatria que participaram do grupo de análise. A primeira reunião, um encontro virtual, com utilização da ferramenta *Google Meet* ^®^, com duração de 120 minutos, contou com a presença de profissionais com experiência em oncologia (enfermeiros, médicos, farmacêuticos e um técnico de informática responsável pelo desenvolvimento do sistema de gestão integrado utilizado na instituição). Houve a discussão, em linhas gerais, dos incidentes identificados e dos processos de cuidado associados. O foco da reunião foi a descrição e compreensão do incidente, mas possíveis ações de melhorias também foram apontadas e registradas.

Posteriormente, duas reuniões presenciais, com duração de 90 minutos, foram organizadas para discussão dos incidentes relativos aos processos de preparo e dispensação, com a presença de cinco farmacêuticos e três técnicos de farmácia e relativos à prescrição médica, com dois médicos e dois farmacêuticos.

Em cada encontro, os participantes utilizaram a técnica de *brainstorming*, que é uma rodada de ideias, em curto período, destinada à busca de sugestões através do trabalho de grupo. O uso desse método baseia-se no pressuposto de que um grupo é capaz de ter mais ideias do que os indivíduos isolados. Tem como finalidade ocasionar o máximo de informações por meio do conhecimento dos membros envolvidos no assunto, buscando encontrar as possíveis causas de um determinado problema^(23)^. Houve a sistematização de ideias utilizando o método dos cinco porquês, orientado pelo questionamento de “Por que este incidente ocorreu?”. A cada resposta descrita, perguntava-se por que a afirmação anterior era verdadeira, até que se cessassem as respostas, sugerindo a identificação da causa raiz. Com base nas repostas relatadas pelos participantes, utilizou-se a ferramenta de diagrama de causa e efeito (diagrama de Ishikawa ou espinha-de-peixe) para exploração e indicação das causas possíveis dos incidentes analisados^(16-17,24)^. Nesses dois encontros, a aplicação da ACR permitiu organizar, classificar, documentar e exibir graficamente as causas dos incidentes, agrupados por categorias, para discussão e análise pelos participantes^(17)^.

## RESULTADOS

No período de 24 meses do estudo, foram registrados 41 incidentes por farmacêuticos e enfermeiros. No ano de 2019, foram atendidos 281 pacientes, gerando 5.127 atendimentos e notificados sete incidentes para seis pacientes. Um incidente relacionava-se ao processo de dispensação (14%), dois, ao processo de preparo (29%), e quatro, ao processo de prescrição (57%), sendo que um atingiu o paciente, mas não gerou dano, ocorrendo a administração de sobredose de quimioterápico.

No ano de 2020, foram atendidos 280 pacientes, com realização de 4.897 atendimentos e notificados 34 incidentes para 30 pacientes por busca ativa, após implantação da planilha de monitoramento. Nenhum incidente atingiu o paciente, demonstrando a efetividade das barreiras presentes. Das 34 notificações, dois incidentes eram de dispensação (6%), 10, de preparo (29%), e 22, de prescrição (65%).

Os incidentes envolvendo a prescrição médica de quimioterapia foram os de maior prevalência. Das 26 notificações durante o período do estudo, 50% eram prescrições manuais, 26,9%, prescrições eletrônicas, e 23,1%, prescrições eletrônicas com alteração manual. Os 22 registros de 2020 foram equivalentes a 1,4% das prescrições analisadas.

O Quadro 1 resume as principais informações dos incidentes encontrados e dos fatores contribuintes identificados pelo grupo multidisciplinar que analisou os incidentes; nesta, destaca-se como mais frequente o medicamento prescrito em dia ou dose diferente do protocolo terapêutico, envolvendo quatro medicamentos.

**Quadro 1:** Incidentes com medicamento antineoplásico injetável e fatores contribuintes nos processos de prescrição, preparo e dispensação no centro de referência estudado (n=41), Rio de Janeiro, Rio de Janeiro, Brasil, 2019-2020

Tipo	Descrição	N	Fatores Contribuintes	Medicamentos Envolvidos
Prescrição	Medicamento prescrito em dia ou dose diferente do protocolo terapêutico	7	Prescrição manual Interrupções Ausência de prescrição eletrônica para alguns Protocolos	Asparaginase, carboplatina Ciclofosfamida, vincristina
	Ajuste da fórmula por peso para paciente com menos de 10 ou 12 Kg	5	Ausência de alerta (barreira) eletrônico	Actinomicina^#^, carboplatina, etoposido, ifosfamida
	Omissão de medicamento de suporte	5	Falta de política para inclusão de medicamentos de suporte Carência de educação permanente para os profissionais	Difenidramina, eletrólitos, hidrocortisona, ondansetrona
	Equívoco no cálculo da dose	4	Ausência de prescrição eletrônica falta de atenção	Ciclofosfamida, eletrólitos
	Necessidade de redução de dose	3	Ausência de alerta (barreira) eletrônico Sobrecarga da equipe	Carboplatina, irinotecano, mesna
	Omissão de dose	1	Ausência de prescrição eletrônica Falta de atenção	Citarabina
	Presença de informação duvidosa	1	Desconhecimento dos protocolos cadastrados	Ciclofosfamida
Preparo	Pedido de preparo do medicamento com dose divergente	5	Equívoco na transcrição Muitas interrupções Ausência de conferência do medicamento manipulado com a prescrição	Carboplatina, etoposido, ifosfamida, mesna, vincristina
	Pedido equivocado de preparo do medicamento não prescrito	4	Erro na transcrição Elevado número de prescrições para analisar Muitas interrupções Ausência de conferência do medicamento manipulado com a prescrição Múltiplas tarefas Demora no deslocamento dos prontuários	Carboplatina, Doxorrubicina, vincristina
	Identificação errada no frasco dos produtos manipulados na cabine de segurança biológica	2	Falta de conferência do rótulo com a bolsa manipulada Horário de entrega das prescrições na farmácia Quantidade de pacientes Espaço físico limitado Déficit de funcionários Sobrecarga de trabalho Cansaço dos funcionários que ficam muito tempo na área de manipulação	Ciclofosfamida/ifosfamida Ciclofosfamida/citarabina
	Rotulagem errada pelo técnico	1	Falta de conferência do rótulo com a bolsa manipulada Déficit de funcionários Sobrecarga de trabalho Cansaço dos funcionários que ficam muito tempo na área de manipulação	Doxorrubicina
Dispensação	Omissão de medicamento prescrito	2	Erro na transcrição Ausência de conferência do medicamento manipulado com a prescrição Múltiplas tarefas	Vincristina, mesna
	Medicamento dispensado para o paciente errado	1	Espaço físico limitado Falta de material de embalagem adequado Rótulo manual com letra ilegível	Ondansetrona

# *- incidente que atingiu o paciente; Kg - quilogramas*.

Os principais fatores contribuintes mencionados pelos participantes, para as três etapas avaliadas, foram agrupados na Figura 2. Assim, esses relacionaram-se a aspectos organizacionais, individuais, além da tarefa, do ambiente e da equipe.

**Figura 2:** Diagrama de causa e efeito dos incidentes com medicamento antineoplásico injetável no centro de referência estudado, Rio de Janeiro, Rio de Janeiro, Brasil, 2019-2020

## DISCUSSÃO

Este estudo permitiu que a equipe responsável pela assistência farmacêutica nas seções de oncologia e hematologia pediátricas pudesse identificar e analisar incidentes relacionados ao uso de antineoplásicos, melhorando a qualidade do cuidado no hospital estudado. Além disso, permitiu a compreensão da complexidade e da abrangência dos erros de medicação ao identificar microprocessos e atividades interligadas e inter-relacionadas envolvendo toda a equipe multidisciplinar, executadas por diversos profissionais. Ademais, as ferramentas analíticas empregadas podem, potencialmente, ser aplicadas a outros problemas na área de qualidade do cuidado, segurança do paciente e outras questões na gestão da organização. Vale destacar o mérito de envolver coletivamente a equipe de profissionais e, assim, melhorar a comunicação e fortalecer a cultura organizacional, em especial a cultura de segurança^(25)^.

Um dos principais eixos para um programa efetivo nessa área são os sistemas de notificações voluntárias, pois podem produzir informação útil e estratégica para garantir a qualidade e fomentar a cultura de segurança. Para que isso ocorra, tais sistemas precisam ter uso disseminado entre os profissionais. Nesse sentido, entende-se que a subnotificação de incidentes compromete a segurança do paciente e é um grande desafio das instituições^(26-27)^. O presente estudo pôde dimensionar o quanto os incidentes podem ser subnotificados. Observou-se uma expressiva diferença entre as fases de coleta de dados. A diferença de notificações foi quase cinco vezes maior na segunda fase (busca ativa), na qual houve um incentivo e estímulo às notificações.

O baixo índice de notificações no período de 2019 corrobora o descrito na literatura, que aponta que um alto percentual de profissionais não notifica eventos ou incidentes relacionados à segurança dos pacientes^(28)^. A falta de notificação, mesmo quando o incidente não atinge o paciente, priva o serviço de saúde de fazer uma análise adequada dos fatores contribuintes, promover intervenções assertivas e melhorar a qualidade do cuidado ao paciente. Bião e Silva^(28)^ debruçaram-se sobre a cultura de segurança do paciente em uma organização hospitalar brasileira de grande porte e utilizando entrevistas com os profissionais de saúde, e encontraram que 87,8% dos profissionais não notificaram nenhum evento em 12 meses. Esses achados sugerem que os profissionais não notificam incidentes por receio de repreensão, punição e humilhação, ou pela descrença de que a notificação não acarretará nenhuma mudança; assim, reforçam que a cultura de segurança justa não está completamente introjetada nas organizações de saúde^(29-30)^.

A prescrição é um estágio crítico no processo de medicação, e um erro de dose nessa etapa acarreta um alto risco de causar danos ao paciente. Erros de prescrição podem ser evitados antes da administração, se as estruturas de segurança apropriadas estiverem instaladas. A padronização de processos, aprimoramento e ênfase à educação dos profissionais prescritores e expansão da atuação dos farmacêuticos clínicos para melhor integração da equipe de saúde são medidas de melhoria apontadas como fatores que minimizam os erros nas prescrições^(31-33)^. A frequência de erros de prescrição em 2020 foi de 1,4%, e esse insere-se na faixa reportada na literatura internacional, que varia entre 0,1% e 24,6%^(34)^. Outro estudo verificou que o erro de medicação mais frequentemente encontrado foi o de prescrição (42,31%), seguido do erro de administração (37,18%)^(35)^. Os principais medicamentos envolvidos foram ciclofosfamida, citarabina, doxorrubicina, etoposido, ifosfamida, irinotecano e vincristina. Na literatura, os medicamentos mais comumente relatados foram metotrexato (15%), citarabina (12%) e etoposídeo (8%)^(32)^.

O programa de computador para gestão de prescrição do hospital, desenvolvido na própria instituição, tem capacidade para extinguir as prescrições manuscritas. A implementação de sistemas de prescrição eletrônica representa um grande avanço dentro das estratégias utilizadas para minimizar erros decorrentes de prescrições, mas, para que isso ocorra, essa medida deve ser adotada por toda a equipe médica. A maneira como as prescrições eram redigidas (manuscritas ou eletrônicas) se mostrou relacionada aos incidentes observados, visto que, entre todos os incidentes de prescrição registrados, 73% envolveram prescrições manuais ou eletrônicas com alguma alteração manual. Albarrak *et al*.^(36)^ observaram que a distribuição dos erros entre as prescrições manuscritas e eletrônicas foi de 35,7% e 2,5%, respectivamente. Estudos evidenciam que a prescrição eletrônica pode reduzir a ocorrência de erros de medicação, melhorando a qualidade da prescrição e a segurança do paciente^(37)^.

Alguns incidentes de prescrição poderiam ser evitados por barreiras, como a emissão de alertas automáticos e apropriados durante o processo de prescrição eletrônica, auxiliando e apoiando o médico na decisão clínica. A adesão de ferramentas informatizadas para a prescrição é estratégica para um processo seguro, sendo indispensável a verificação das características operacionais do sistema, pois a existência de um programa para a prescrição de medicamentos não credencia o processo como seguro^(38-39)^.

Reinhardt *et al*.^(31)^ relatam que o aprimoramento tecnológico favorece um potencial aumento da segurança no uso de quimioterapia, mas, mesmo com modificações no programa de computador, cerca de 39% dos erros permaneceriam não identificados e não corrigidos pelo sistema informatizado. Portanto, os profissionais envolvidos na cadeia continuam a ser de fundamental importância na interceptação de tais erros, contribuindo assim para melhorar a segurança dessa população de pacientes de alto risco.

Partindo da premissa de que a ocorrência de erro de medicação pode se concretizar em qualquer fase do processo, que esse é comedido de decisões e ações de diversos profissionais com variadas formações e comportamentos, torna-se incontestável a sensibilização dos profissionais passíveis de cometerem erros. Nessa sensibilização, enfatizam-se a necessidade de compreensão e a assimilação acerca dos impactos negativos para o processo e riscos para o paciente.

Revisão integrativa identificou que os principais fatores de risco relacionados ao preparo de medicamentos endovenosos eram os aspectos psicológicos e sobrecarga de trabalho, fatores relacionados ao ambiente de trabalho e ao déficit de atualização em educação em saúde^(40)^. Outro estudo descritivo transversal recrutou enfermeiros de três hospitais terciários na Coreia do Sul. Os dados coletados via questionários estruturados mostraram que maiores competências em segurança do paciente estavam diretamente associadas aos profissionais que trabalham menos de 40 horas semanais, enquanto aqueles que trabalham mais de 50 horas semanais apresentaram mais resultados adversos, tendo efeitos prejudiciais na segurança do paciente^(41)^.

As diversas interrupções durante a execução de suas tarefas também foram um ponto crítico levantado no presente estudo. Essas interrupções costumam ser frequentes e enraizadas na cultura das instituições de saúde. Grande parte dos profissionais é condescende, pois considera esse problema como algo inerente ao trabalho^(42)^. Esse cenário favorável à ocorrência de incidentes na assistência, que impactam na precisão das atividades realizadas, foi atribuído como um fator contribuinte para a ocorrência de incidentes. De acordo com as recomendações do Instituto para Práticas Seguras no Uso dos Medicamentos^(42-43)^, é imprescindível o planejamento de estratégias para evitar interrupções de atividades durante o processo de medicação. Vale ressaltar que as estratégias serão mais satisfatórias se acompanhadas de uma abordagem educativa que promova a conscientização dos profissionais e a mudança de cultura na instituição. Sob outra perspectiva, equipes com número de profissionais reduzido e inadequado, quando comparadas ao número de pacientes em atendimento, também estão mais propícias a serem interrompidas, uma vez que se mostram mais exaustas, precipitadas e com demanda de múltiplas tarefas^(42)^.

Nesse contexto, a gestão de recursos humanos desempenha um papel fundamental nos serviços de saúde, equilibrando as necessidades da instituição com o uso adequado dos recursos financeiros e bem-estar dos profissionais e pacientes, afetando diretamente a qualidade dos serviços prestados e o grau de satisfação dos usuários^(44)^. As unidades de saúde devem contar com profissionais na quantidade adequada, proporcionando o desenvolvimento de processos seguros e sem sobrecarga ocupacional, respeitando o limite de carga-horária semanal legalmente estabelecida e a legislação vigente^(42,44)^.

É importante ressaltar que apenas um dos incidentes registrados chegou ao paciente e, apesar do potencial, não causou dano grave, pois o paciente foi monitorado após a identificação do evento. Isso reflete a importância do cuidado colaborativo realizado pela equipe multidisciplinar na promoção da qualidade e segurança do paciente. Watts e Parsons^(45)^ mostraram que uma equipe multidisciplinar, trabalhando em um sistema prospectivo de vigilância farmacêutica de erros de prescrição e administração de quimioterapia, conseguiu reduzir pela metade a ocorrência de erros observados em oncopediatria. Assim, no nosso entendimento, esse estudo reforça (i) a importância dos microprocessos que envolvem a assistência farmacêutica e (ii) como uma análise multidisciplinar e minuciosa pode revelar caminhos de melhorias na qualidade do cuidado.

### Limitações do estudo

Este estudo possui limitações, entre as quais destaca-se o desenho de estudo de caso único, que se debruçou sobre a experiencia de um hospital de referência em oncologia pediátrica. O número de reuniões realizadas (três) mostrou-se adequado à proposta, mas é possível que a ampliação do número de encontros poderia ter propiciado maior exploração e discussão exaustiva dos incidentes encontrados, aspecto aqui não avaliado, pois deteve-se sobre os fatores contribuintes. Sabe-se que a ACR é uma estratégia de fácil utilização em hospitais, útil para a identificação de causas remotas e imediatas de incidentes de segurança; contudo, a efetividade na implementação de medidas para prevenção de problemas envolve outros elementos do contexto organizacional interno e externo^(46)^.

### Contribuições para a área da enfermagem, saúde ou políticas públicas

Devido à falta de estudos sobre o cuidado pediátrico, um ponto forte deste estudo é a possibilidade de utilizar os dados analisados para a comparação de resultados com outras instituições e sensibilização de profissionais quanto à cultura de segurança no uso de medicamentos antineoplásicos, tendo em vista que a metodologia empregada poderia ser adaptada para subsidiar a mesma análise em outras instituições.

## CONCLUSÕES

Os achados evidenciaram a magnitude da ocorrência de incidentes de prescrição, seguida pelas ocorrências em preparo de medicamentos. Identificou-se que os principais fatores que contribuíram para a ocorrência de incidentes de segurança foram a ausência de conferência e de barreiras eletrônicas, elaboração de prescrições e rótulos manuais, questões organizacionais e ambientais, problemas de comunicação, sobrecarga de trabalho, fadiga e falta de atenção Os resultados apresentados mostram que uma intervenção relativamente simples, como registro adequado e aplicação da ACR aos incidentes identificados, pode proporcionar uma melhoria na qualidade do cuidado em oncopediatria, mapeando os fatores contribuintes e possibilitando que os gestores desenvolvam um plano de ação efetivo para mitigar os riscos associados às etapas analisadas.

Espera-se que a experiência descrita na aplicação da ACR e os resultados obtidos possam servir de subsídios para melhorar a segurança do paciente, estimulando e norteando gestores, autoridades e profissionais de saúde no que diz respeito à proposição de iniciativas e estratégias que melhor respondam às necessidades especificas das instituições. A implantação de processos seguros sistemáticos no uso do medicamento não representa garantia de segurança e qualidade da assistência à saúde apenas para pacientes, significando também a proteção dos profissionais e instituições envolvidas.

## Data Availability

https://doi.org/10.48331/scielodata.ZOQGJE
